# Coronavirus Disease (COVID-19) Outbreak: Hypofractionated Radiotherapy in Soft Tissue Sarcomas as a Valuable Option in the Environment of Limited Medical Resources and Demands for Increased Protection of Patients

**DOI:** 10.3389/fonc.2020.00993

**Published:** 2020-06-05

**Authors:** Mateusz Jacek Spałek, Piotr Rutkowski

**Affiliations:** Department of Soft Tissue/Bone Sarcoma and Melanoma, Maria Sklodowska-Curie National Research Institute of Oncology, Warsaw, Poland

**Keywords:** sarcoma, hypofractionation, perioperative radiotherapy, COVID-19, multidisciplinary treatment, rare cancers

## Introduction

The coronavirus disease (COVID-19) pandemic in 2019/2020 became a significant problem not only for first-line healthcare but also for cancer patients, who are at the risk of severe or fatal outcome of potential infection. In recently published papers about radiotherapy (RT) in various cancers, there are suggestions to use more hypofractionated RT (HFRT) regimens during the COVID-19 pandemic to reduce overall treatment time (1). Unfortunately, HFRT in soft tissue sarcomas (STS) is rarely mentioned and underestimated. SARS-CoV-2 infection in a tertiary STS clinic or RT department might cause a shortage of experienced staff by putting them in quarantine. What is more, the interrupted treatment cannot be continued in other institutions with the maintenance of high-quality care due to lack of necessary knowledge, experience, and equipment. Perioperative conventionally fractionated RT (CFRT), namely between 1.8 and 2.0 Gy per fraction by 5 to 7 weeks, is considered to be a standard regimen in STS (2). There is growing evidence that preoperative HFRT could be also a possible therapeutic approach and its wide introduction may be a controversial but necessary solution.

## Rationale for Preoperative Approach

Preoperative RT in STS is not widely accepted due to the higher risk of postoperative wound complications. However, wound complications, even serious, are usually manageable and reversible, while late toxicity, manifested as complications related to fibrosis, is commonly permanent and can lead to severe impairment of patient's function and quality of life. In a phase III randomized clinical trial that compared preoperative and postoperative RT in STS, wound complications occurred in 35% of patients in the preoperative group and in 17% in the postoperative group (3). After prolonged follow-up, late toxicity was observed more frequently in the postoperative arm than in the preoperative arm without any significant differences in local control and survival (4). The preoperative RT has more advantages i.e., visible tumor volume, less healthy tissues within irradiated volume, lower total dose, better tissue oxygenation, and lower risk of tumor cell seeding during surgery. Moreover, preoperative RT may provide substantial benefit for patients with locally advanced disease, allowing conservative or limb-sparing surgery in marginally-resectable or unresectable STS (5). Finally, cost-effectiveness analysis supports preoperative RT in STS (6).

## Rationale for Hypofractionation

HFRT has a clinical rationale. HFRT regimens could significantly shorten overall treatment time. Decreased exposure to potential SARS-CoV-2 infection in a hospital as well as compliance with treatment, convenience and cost favor HFRT. Additionally, HFRT has a radiobiological rationale. Basing on the linear-quadratic model, a larger dose per fraction applied to tumors with a lower α/β ratio should result in better tumor control. Heterogeneity of STS translates into a wide spectrum of radiosensitivity, however, for most STS subtypes α/β ratio is considered as lower than 10 Gy (7). For example, calculated liposarcoma and rhabdomyosarcoma α/β ratios were as low as 0.4 and 2.8 Gy, respectively (8). Furthermore, assuming low α/β ratio for STS and better responsiveness to a larger fraction size, HFRT may allow de-escalation of total dose with constant tumor control. That may result in decreased toxicity from surrounding tissues. For purposes of comparison of the different fractionation schedules in this review, the equivalent dose in 2-Gy fractions (EQD2) was calculated assuming the α/β ratio for STS of 4 Gy, as in calculations performed in other studies (2, 7). The results were presented in Table 1.

**Table 1:** Preoperative radiotherapy regimens in soft tissue sarcomas in major published studies.

	**References**	**Evidence**	**N of patients**	**Dominant preoperative regimen**	**EQD2 α/β 4 Gy**	**Tumors >10 cm**	**Surgery after RT**	**R0**	**@years local control**	**All wound complications @severe^∧^**	**Reported late toxicity**	**@years estimated survival**
Conventionally fractionated RT regimens	Pollack et al. (9)	Retrospective cohort	128 (preop) 51 CHT+RT 77 RT	CHT^*^^&^50 Gy/25 fr.	50 Gy	ND median 10 cm	Delayed	92%	@5y 82%	25%@ND	6%	ND
	O'Sullivan et al. (3, 10) Davis et al. (4)	Phase III RCT (preop vs. postop)	94 (preop)	50 Gy/25 fr.	50 Gy	35%	Delayed (3–6 weeks)	84%	@5y 93%	35%@17%	G2+: fibrosis 32% JS 18% edema 15%	@5y DRFS 67% OS 73%
	Zagars et al. (11)	Retrospective cohort	271 (preop) 179 CHT+RT 92 RT	CHT^*^^&^ 50 Gy/25 fr.	50 Gy	42%	Delayed (4–6 weeks)	86%	@5y 85% @10y 83%	ND	5%	@5y DRFS 64% @10y DRFS 61% DSS 64%
	Hui et al. (12)	Retrospective cohort	67	50.4 Gy/28 fr.	48.7 Gy	ND median 6 cm	Delayed (3–6 weeks)	99%	@5y 93%	41%@18%	7%	@5y DRFS 68% OS 73%
	Kraybill et al. (13)	Phase II single arm CT	64	MAID 22 Gy/11 fr. MAID 22 Gy/11 fr. MAID	44 Gy	ND median 15 cm	Delayed	91%	@3y 90%	11%@3%	ND	@3y DRFS 65% OS 75%
	Canter et al. (14)	Retrospective cohort	25	50 Gy/25 fr.	50 Gy	36%	Delayed (4–6 weeks)	84%	@3y 100%	28%@16%	ND	ND
	Yoon et al. (15)	Phase II single arm CT	20	Bevacizumab 50.4 Gy/28 fr.	48.7 Gy	ND median 8 cm	Delayed (6–7 weeks)	ND	@2y 95%	20%@ND	ND	@2y DRFS 65%
	Shah et al. (16)	Retrospective cohort	30	50 Gy/25 fr.	50 Gy	40%	Delayed (4–6 weeks)	ND	@5y 100%	23%@20%	ND	@5y DRFS 61% OS 69%
	O'Sullivan et al. (17)	Phase II single arm CT	59	50 Gy/25 fr.	50 Gy	ND median 10 cm	Delayed	93%	@5y 88%	31%@10%	Moderate: skin 2% fibrosis 9% JS 7% edema 11%	@5y DRFS 67% OS 75%
	Lewin et al. (18)	Phase Ib/II single arm CT	9	Sunitinib 50.4 Gy/28 fr.	48.7 Gy	ND median 10 cm	Delayed (3–6 weeks)	ND	ND	ND	Any G: 78%	@2y PFS 44% OS 56%
	Canter et al. (19)	Phase I single arm CT	8	Sorafenib 50 Gy/25 fr.	50 Gy	63%	Delayed (4–6 weeks)	75%	@3y 100%	38%@ND	ND	@3y DRFS 42% OS 75%
	Wang et al. (20)	Phase II single arm CT	79	50 Gy/25 fr. with reduced margins	50 Gy	ND median 11 cm	Delayed	76%	@2y 94%	37%@25%	G2+: fibrosis 5% JS 4% edema 5%	@2y DRFS 65% OS 81%
	Haas et al. (21)	Phase I single arm CT	11	Pazopanib 50 Gy/25 fr.	50 Gy	27%	Delayed (5–7 weeks)	ND	@2y 91%	20%@0%	ND	@2y DRFS 82%
	Jakob et al. (22)	Phase Ib/II single arm CT	5	Sunitinib 50.4 Gy/28 fr.	48.7 Gy	40%	Delayed (5–8 weeks)	100%	@2y 80%	56%@22%	ND	@2y DRFS 60%
Hypofractionated RT regimens	Temple et al. (23)	Prospective register	42	Doxorubicin 30 Gy/10 fr.	35 Gy	ND	Delayed (4–6 weeks)	ND	@5y 97%	15%@ND	ND	@5y OS 79%
	Ryan et al. (24)	Retrospective cohort	25	EI 28 Gy/8 fr.	35 Gy	ND median 10 cm	Delayed (4–5 weeks)	88%	@2y 88%	ND@20%	ND	@2y DRFS 78% OS 84%
	MacDermed et al. (25)	Retrospective cohort	34 included 6 patients with DM	Ifosfamide 28 Gy/8 fr.	35 Gy	32% (>12 cm)	delayed (4–8 weeks)	100%	@5y 89%	ND@17%	Fibrosis 14% edema 17%	@5y (no DM) DRFS 53% OS 45%
	Meyer et al. (26)	Phase I single arm CT	16 included 2 patients with DM	Sorafenib EI 28 Gy/8 fr.	35 Gy	ND	Delayed	94%	@2y 100%	38%@ND	ND	@2y PFS 86%
	Koseła-Paterczyk et al. (27)	Prospective register	272 61 CHT+RT 211 RT	CHT^*^^&^ 25 Gy/5 fr.	37.5 Gy	42%	Immediate (3–7 days)	79%	@3y 81%	all 32% @12% 53% CHT+RT 53% @21% RT 27% @9%	15% all 23% CHT+RT 12% RT	@5y OS 60%
	Pennington et al. (28)	Retrospective cohort	116	CHT^*^ 28 Gy/8 fr.	35 Gy	47%	Delayed (2–3 weeks)	93%	@3y 89% @6y 83%	10%@1%	4%	@3y DRFS 75% OS 82% @6y DRFS 65% OS 67%
	Spalek et al. (29)	Phase II single arm CT	29 MLPS only	25 Gy /5 fr.	37.5 Gy	66%	Delayed (6–8 weeks)	93%	@1y 100%	31%@ND	ND	@1y DRFS 86%
	Spalek et al. (5)	Phase II single arm CT	30 marginally resectable or unresectable	1x AI 25 Gy/5 fr. 2x AI	37.5 Gy	74%	Delayed (6–8 weeks)	73%	@1y 97%	23%@7%	ND	@1y DRFS 74%
	Parsai et al. (30)	Retrospective cohort	16 3 CHT+RT 13 RT	CHT^*^ 30 Gy /5 fr.	50 Gy	25%	Immediate (0–7 days)	63%	@1y 100%	31%@19%	ND	ND
	Kalbasi et al. (31)	Phase II single arm CT	50	30 Gy/5 fr.	50 Gy	24%	Delayed (2–6 weeks)	82%	@2y 94%	32%@24%	G1: fibrosis 24% JS 11% edema 4% G2: fibrosis 11% JS 11% edema 4%	@2y DRFS 79%

*AI, doxorubicin, ifosfamide; EI, epirubicin, ifosfamide; CHT, chemotherapy; CT, clinical trial; DM, distant metastases; DRFS, distant recurrence-free survival; DSS, disease-specific survival; EQD2, equivalent dose in 2-Gy fractions; G, grade; JS, joint stiffness; MAID, mesna, doxorubicin, ifosfamide, dacarbazine; MLPS, myxoid liposarcomas; ND, no data; OS, overall survival; PFS, progression-free survival; RCT, randomized clinical trial; RT, radiotherapy; STS, soft tissue sarcomas*.

* *various regimens were used*.

& *only part of a group received chemotherapy*.

∧ *assessed by authors as grade 3 or higher, or requiring reoperation*.

## Available Evidence and HFRT Regimens

Preoperative HFRT in STS has been validated in retrospective analyses, prospective registries, and phase I-II clinical trials (5, 23–31). However, randomized phase III trials comparing preoperative CFRT with HFRT are lacking. As with CFRT, HFRT could be combined with systemic treatment (2). Despite scarce evidence on the efficacy of perioperative chemotherapy in STS, it is commonly applied as a part of treatment. A combination of RT with targeted therapy is still under investigation giving promising results but also unexpected toxicities (18, 21). The investigated regimens of preoperative RT in STS were summarized in Table 1 (3–5, 9–31). Presented data should be interpreted with caution because analyzed populations were not comparable as they differed with many factors including patients' characteristics, STS subtypes, tumor size, indications for RT, RT techniques, elective margins, and quality of surgery. Nevertheless, the results of HFRT regimens seem very similar to those of CFRT regimens. The 5-year local control was 82–100% (median 91%) in CFRT and 89 and 97% in two studies on HFRT. Furthermore, the rate of severe wound complications was 0–25% (median 17%) in CFRT and 1–24% (median 18%) in HFRT. It is noticeable, that EQD2 is lower than 50 Gy in the majority of analyzed HFRT regimens.

## Discussion

Available data suggest that preoperative HFRT in STS is a promising treatment option providing satisfactory local control with acceptable toxicity. Nevertheless, it has been not widely adapted in clinical practice. COVID-19 pandemic may be the appropriate time to rethink RT in STS.

Routine use of preoperative HFRT may be limited by some concerns. One may fear that decreased EQD2 in preoperative HFRT will result in worse local control. However, the current standard of 50 Gy in 2-Gy fractions is not based upon strong evidence coming from randomized clinical trials with various dose levels or fractionation regimens. In the analysis performed by Haas et al. it has been shown that dose-response relationship for local control in preoperative RT is clear only below 28 Gy in 8 fractions of 3.5 Gy (EQD2 = 35 Gy if α/β = 4 Gy) (2). Above that level, the benefit in local control from increased total dose may be negligible, especially when RT is combined with preoperative chemotherapy or targeted therapy. Data presented in the Table 1 suggest that this assumption may be correct because local control in all described regimens is higher than 80% despite various EQD2. Interestingly, HFRT regimen described by Koseła-Paterczyk et al. (25 Gy in 5 fractions) given in the majority of patients without preoperative chemotherapy provided lower (but acceptable) local control than regimens with higher fraction and total doses (30 Gy in 5 fractions) or one with the same fractionation regimen but combined with sequential anthracycline-based chemotherapy (5, 27, 30, 31). The same 5 × 5 Gy regimen without chemotherapy but with delayed surgery resulted in 100% 1-year control rate in patients with myxoid liposarcomas that are considered radiosensitive (29).

Furthermore, the preoperative approach and hypofractionation in STS remain controversial due to the risk of treatment-related morbidity. Wound complications are serious adverse effects of any preoperative RT in STS. However, this toxicity could be predicted by assessment of patient-related risk factors, such as smoking, diabetes, obesity, and tumor location (lower limbs) (32). Although larger doses per fraction could theoretically increase the risk of late toxicities, such assumption was neither confirmed in clinical trials with preoperative and definitive HFRT in other neoplasms, i.e., rectal, prostate or lung cancer, nor in presented data regarding HFRT in STS. Moreover, the occurrence of selected late toxicities after combined treatment of STS could be predicted and often reduced. For example, periosteal location of tumor, higher mean and maximal dose to bone as well as volume of bone irradiated to over 40 Gy in 2-Gy fractions increase the risk of pathologic fractures (33). Proper treatment planning and choice of RT techniques with intensity modulation can significantly reduce both early and late toxicity (17, 20). Thus, taking into account local control and toxicity, the choice of RT regimen should be based on several factors, i.e., patients' characteristics, tumor location and size, STS subtype and its radiosensitivity, risk of local and distant relapse, availability of equipment, RT techniques, and systemic treatments.

No direct comparison of preoperative CFRT and HFRT regimens in STS was performed in the literature. While randomized clinical trials are still the gold standard, other approaches when investigating various treatments for rare diseases should be considered, such as Bayesian trial design. Moreover, after discussion within the multidisciplinary tumor board, the individualized treatment regimens may be proposed to patients then collected in prospective registries.

Radiation oncologists are not front-line fighters in COVID-19 times, but they can deal with the spread of infection another way. In a global emerging situation of COVID-19 pandemic, the benefits of preoperative HFRT for STS patients may outweigh risks. Besides good efficacy and acceptable toxicity, HFRT decreases the hospital-associated COVID-19 infection risk, as well as the risk of treatment interruption, delay, or its poor quality if performed outside STS tertiary center. Available treatment options and concerns should be discussed with the patient in a shared decision-making process.

## Author Contributions

MS and PR conceived of the presented idea and discussed the concerns and contributed to the final manuscript. MS performed the literature review and prepared the draft. PR provided critical revision of the article.

## Conflict of Interest

The authors declare that the research was conducted in the absence of any commercial or financial relationships that could be construed as a potential conflict of interest.
